# Impact of Hospital Bed Shortages on the Containment of COVID-19 in Wuhan

**DOI:** 10.3390/ijerph17228560

**Published:** 2020-11-18

**Authors:** Weike Zhou, Aili Wang, Xia Wang, Robert A. Cheke, Yanni Xiao, Sanyi Tang

**Affiliations:** 1 School of Mathematics and Information Science, Shaanxi Normal University, Xi’an 710062, China; wkzhou@snnu.edu.cn (W.Z.); xiawang@snnu.edu.cn (X.W.); 2 School of Mathematics and Information Science, Baoji University of Arts and Sciences, Baoji 721013, China; wangaili@bjwlxy.edu.cn; 3 Natural Resources Institute, University of Greenwich at Medway, Central Avenue, Chatham Maritime, Kent ME4 4TB, UK; r.a.cheke@greenwich.ac.uk; 4 School of Mathematics and Statistics, Xi’an Jiaotong University, Xi’an 710049, China; yxiao@mail.xjtu.edu.cn

**Keywords:** COVID-19 outbreak, transmission model, hospital beds, effective reproduction number, sensitivity analysis

## Abstract

The global outbreak of COVID-19 has caused worrying concern amongst the public and health authorities. The first and foremost problem that many countries face during the outbreak is a shortage of medical resources. In order to investigate the impact of a shortage of hospital beds on the COVID-19 outbreak, we formulated a piecewise smooth model for describing the limitation of hospital beds. We parameterized the model while using data on the cumulative numbers of confirmed cases, recovered cases, and deaths in Wuhan city from 10 January to 12 April 2020. The results showed that, even with strong prevention and control measures in Wuhan, slowing down the supply rate, reducing the maximum capacity, and delaying the supply time of hospital beds all aggravated the outbreak severity by magnifying the cumulative numbers of confirmed cases and deaths, lengthening the end time of the pandemic, enlarging the value of the effective reproduction number during the outbreak, and postponing the time when the threshold value was reduced to 1. Our results demonstrated that establishment of the Huoshenshan, Leishenshan, and Fangcang shelter hospitals avoided 22,786 people from being infected and saved 6524 lives. Furthermore, the intervention of supplying hospital beds avoided infections in 362,360 people and saved the lives of 274,591 persons. This confirmed that the quick establishment of the Huoshenshan, Leishenshan Hospitals, and Fangcang shelter hospitals, and the designation of other hospitals for COVID-19 patients played important roles in containing the outbreak in Wuhan.

## 1. Introduction

A severe outbreak of coronavirus disease (COVID-19) has spread globally and caught the eyes of the world in 2020. The virus that is responsible for COVID-19 is a new strain of coronaviruses that has not been previously identified in humans, and was named severe acute respiratory syndrome coronavirus 2 (SARS-CoV-2) [1,2,3]. The rapidly increasing number of reported cases reveals that the novel virus is easily spread between people [4]. On 11 March 2020, as the virus spreads increasingly worldwide, the World Health Organization (WHO) designated the COVID-19 outbreak as a “pandemic” [1].

As of 23 October 2020, there have been 41,104,946 confirmed cases with 1,128,325 deaths reported worldwide [1]. The alarming levels of spread and severity of the disease have posed a burden on the development of nations’ economies and societies. The number of confirmed cases and deaths worldwide are still climbing rapidly, despite the many different preventive measures implemented to contain or mitigate the transmission of COVID-19. One of the main reasons why the spread of COVID-19 is out of control in many countries is the shortage of medical resources, which may lead to many more infections due to the incomplete isolation of undetected or non-hospitalized patients. Thus, examining the impact of medical resources is an important issue for the control of COVID-19 outbreaks, the subject of our study.

As with many other countries, medical resources in Wuhan, which was the first city in China that reported information about COVID-19, were initially in short supply. The media reported a significant shortage of hospital beds during the early phase of the COVID-19 outbreak in Wuhan, even though a series of prompt public health measures such as contact tracing, quarantine, travel restrictions, and lock-down of cities had been strictly carried out to prevent the spread of the disease since 23 January 2020 [4]. To meet an urgent need of medical resources, the public health authorities mobilized health-care workers from other provinces of China to give assistance, and medical resources were delivered from various regions to Wuhan to relieve the burden of medical-resource constraints. However, it was still far from sufficient for the very severe situation in Wuhan. Further, to make sure medical facilities for COVID-19 available to all people needing medical assistance [5], a number of hospitals were successively requisitioned as designated hospitals and the Huoshenshan, Leishenshan hospitals and Fangcang shelter hospitals were built for the treatment of confirmed patients [6]. Finally it was reported that all people needing medical assistance were admitted to medical facilities in mid-February [7]. With sufficient medical resources and strict control measures, the outbreak of COVID-19 in Wuhan was contained. Thus, in order to illustrate the important role of timely supplementation of sufficient hospital beds, in this paper we use the example of Wuhan to provide suggestions for policy-makers in other countries.

Many studies have focused on how COVID-19 spreads and on prevention and control strategies from different perspectives. Dynamical models have been used as one of the most useful tools for revealing the transmission mechanisms, estimating the transmission risks, predicting the timing of peaks and the final extent of COVID-19, and for evaluatung the efficacy of non-pharmaceutical interventions, such as travel restrictions, social distancing, lockdown of cities, closing schools, and wearing of face coverings and masks [8,9,10,11,12,13,14]. For example, Wu et al. [8] estimated the basic reproduction number and the size of the epidemic in Wuhan and forecasted the extent of national and global spreads of COVID-19 by using an SEIR metapopulation model. Tang et al. [9] proposed a deterministic model that is based on the clinical progression and effects of strong intervention measures, such as contact tracing, followed by quarantine and isolation. They showed a much higher basic reproduction number to give warning of the high transmissibility of COVID-19 and showed that intensive contact tracing can effectively reduce the potential and severity of the outbreak in Wuhan. Hao et al. [10] highlighted two key features of the outbreak: high covertness and high transmissibility through retrospective analysis. They constructed their SEPHIRE model in order to delineate the full dynamics of COVID-19 in Wuhan and observed that multipronged interventions had significant positive effects on controlling the outbreak, decreasing the reproduction number, and reducing the total number of infections. However, to the authors’ best knowledge, few modelling studies [15,16,17] have examined the role of limited medical resources on the spread of COVID-19. Rong et al. [15] developed an SEIR model that distinguishes between the general population and healthcare staff and studied the effects of healthcare staff on the transmission of COVID-19, when considering that limitation of medical resources would pose a threat not just to patient care, but also to the safety of healthcare staff. Sun et al. [16] described supplementing medical resources by increasing the confirmation rate and the removed rate based on a modified SEIR model. Li et al. [17] treated health-care personnel separately from the general public and constructed several phase-based models in order to study the effect of Fangcang shelter hospitals on the control of the epidemic.

Piecewise smooth models, which are always composed of several different ODE models, can be used to describe the limitation of medical resources by designing a threshold policy [18,19,20,21]. Previous studies mainly focused on theoretical analysis of piecewise smooth models and seldom applied them to an actual problem combined with data. However, data on the density of inpatient beds are an indicator for evaluating the level of health service delivery and, so, the number of available hospital beds can be used in order to assess the availability of medical resources for outbreaks of COVID-19. Hence, in this paper, we quantify variation of medical resources and its influence on the control of the COVID-19 outbreak in Wuhan by using a mathematical modelling method to give greater insight into the impact of hospital bed numbers, their supply rate and the timing of bed supplies on the COVID-19 outbreak in Wuhan. A piecewise smooth model (2) is proposed in order to examine the effect of hospital beds on the containment of COVID-19 explicitly. Time-dependent parameters are adopted to describe the strengthening of control measures and improvements in detection technology, together with an implicit function in terms of the number of hospital beds ($\min\{\eta P,H_{c}\left(t\right)-H\}$) introduced to our targeted model in order to describe the number of newly hospitalized cases in relation to medical-resources constraints.

The rest of the paper is organized, as follows. In Section 2, we introduced the data sets that were used in this study, the piecewise smooth model describing the limitation of hospital beds and the nonlinear least-square method. In Section 3, the parameter values are estimated and the impact of hospital bed numbers, their supply rate, and the timing of when hospital beds are supplied are explored. In Section 4, the results and limitations of the paper were discussed and conclusions were given in Section 5.

## 2. Methods

### 2.1. Data Collection and Analysis

**Figure 1:**
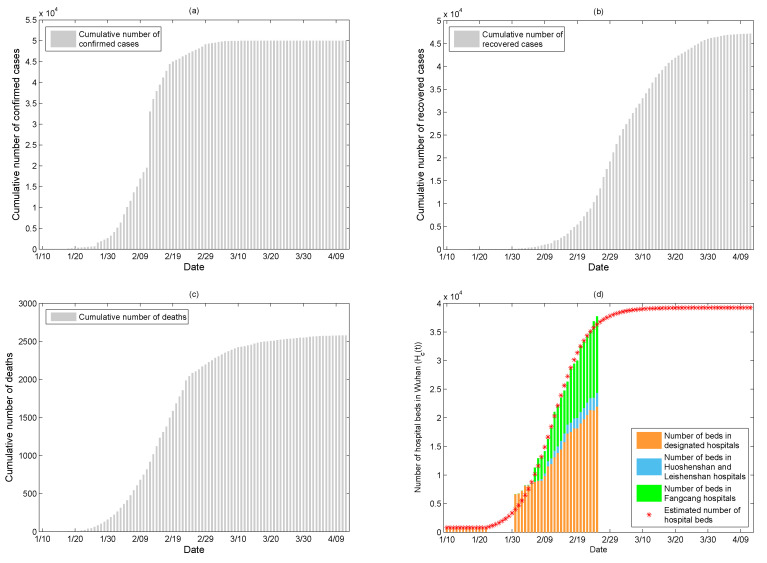
The data on COVID-19 in Wuhan from 10 January to 12 April. (**a**) Cumulative number of confirmed cases; (**b**) cumulative number of recovered cases; (**c**) cumulative number of deaths; and, (**d**) number of hospital beds.

We obtained the data on COVID-19 cases in Wuhan city from 10 January to 12 April 2020, from the National Health Commission of the People’s Republic of China [22], Health Commission of Hubei Province [23], and Wuhan Municipal Health Commission [24]. The data include the number of cumulative confirmed cases (Figure 1a), the number of cumulative recovered cases (Figure 1b), and the number of cumulative deaths (Figure 1c). Note that the data reported were adjusted on 12 February, some COVID-19 induced deaths reported before were cancelled after verification, such that the number of cumulative deaths reported on 13 February (1016 cases) was lower than that on 12 February (1036 cases). Thus, we regenerate the number of cumulative deaths on 12 February by averaging the numbers that were reported on 11 and 13 February.

We also collected information on the number of hospital beds in Wuhan. There were two designated hospitals with about 800 beds for COVID-19 patients before 23 January 2020, when hospital beds were gradually being expropriated for COVID-19 patients. The number of designated hospital beds from 31 January to 25 February (orange bars in Figure 1d) and the number of hospital beds in Huoshenshan and Leishenshan hospitals (blue bars in Figure 1d) were collected from the Wuhan Municipal Health Commission [25], the number of Fangcang shelter hospital beds from 5 to 25 February (green bars in Figure 1d, note that Fangcang shelter hospitals were implemented for the first time on 5 February) were extracted from the reference [6]. Subsequently, we assumed that the number of hospital beds $H_{c}\left(t\right)$ is a constant $H_{c_{0}}$ before 23 January, and it is an increasing function that satisfies the following logistic model (1) from then on. In model (1), *r* is the net increasing rate of hospital beds and *M* is the maximum number of hospital beds in Wuhan during the COVID-19 outbreak. Figure 1d shows the estimated number of hospital beds every day.

(1) $$\begin{array}{c} \frac{dH_{c}\left(t\right)}{dt}=rH_{c}\left(t\right)\left(1-\frac{H_{c}\left(t\right)}{M}\right). \end{array}$$

### 2.2. The Model

**Figure 2:**
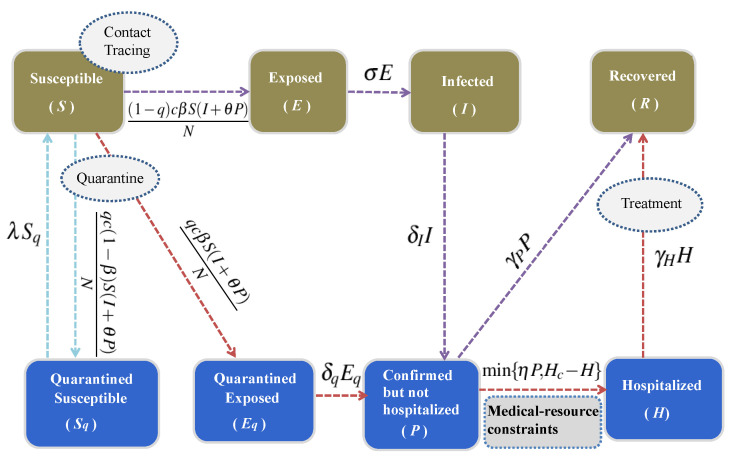
Flow diagram to illustrate the infection dynamics of COVID-19 in Wuhan city. Integrated control measures including intensive contact tracing, quarantine and isolation are illustrated.

Based on the disease progression and intervention measures, we extended the basic SEIR model [8,9] by considering contact tracing, followed by quarantine and hospitalization strategies. The total population *N* is divided into eight compartments, including susceptible (*S*), quarantined susceptible ($S_{q}$), exposed (*E*), quarantined exposed ($E_{q}$), infected (*I*), confirmed but not hospitalized yet (*P*), confirmed and hospitalized (*H*), and recovered (*R*). Subsequently, we have the following model according to the policy of one patient one bed, which means that each hospitalized individual (*H*) would occupy a single hospital bed. See the flow diagram of the model presented in Figure 2.

In model (2), the contact rate is denoted by c(t) and the transmission probability per contact is β, q(t) is the quarantined proportion of individuals exposed to the virus. According to whether the quarantined people that are exposed to the virus ($\frac{q(t)c(t)S(I+\theta P)}{N}$) are effectively infected, they can either move to the quarantined exposed compartment $E_{q}$ ($\frac{q(t)c(t)\beta S(I+\theta P)}{N}$) with transmission probability β, or move to the quarantined susceptible compartment $S_{q}$ ($\frac{q(t)c(t)(1-\beta )S(I+\theta P)}{N}$) with a probability of 1−β. While individuals exposed to the virus missing from the contact tracing ($\frac{\left(1-q(t))c(t)S(I+\theta P\right)}{N}$), will similarly move to the exposed compartment *E* ($\frac{\left(1-q(t))c(t)\beta S(I+\theta P\right)}{N}$) with effective transmission probability β, or just stay in the susceptible compartment *S* ($\frac{\left(1-q(t))c(t)(1-\beta )S(I+\theta P\right)}{N}$) with probability 1−β. 1/σ is the incubation period, λ is the release rate of quarantined susceptible individuals, $\delta_{I}\left(t\right)$ and $\delta_{q}\left(t\right)$ are the diagnosis rates for infected and quarantined exposed individuals, respectively, $\alpha_{I}$, $\alpha_{P}$, $\alpha_{H}\left(t\right)$ are the disease-induced death rates for *I*, *P* and *H*, respectively, η is the hospitalization rate, and $\gamma_{P}$ and $\gamma_{H}\left(t\right)$ are the recovery rates for *P* and *H*, respectively.

(2) $$\begin{array}{c} \left\{\begin{array}{cc} & S^{\prime }=-\frac{\left(c(t)\beta +q(t)c(t)(1-\beta ))S(I+\theta P\right)}{N}+\lambda S_{q},\\ & E^{\prime }=\frac{\left(1-q(t))c(t)\beta S(I+\theta P\right)}{N}-\sigma E,\\ & I^{\prime }=\sigma E-\alpha_{I}I-\delta_{I}\left(t\right)I,\\ & S_{q}^{\prime }=\frac{q(t)c(t)(1-\beta )S(I+\theta P)}{N}-\lambda S_{q},\\ & E_{q}^{\prime }=\frac{q(t)c(t)\beta S(I+\theta P)}{N}-\delta_{q}\left(t\right)E_{q},\\ & P^{\prime }=\delta_{I}\left(t\right)I+\delta_{q}\left(t\right)E_{q}-\min\left\{\eta P,H_{c}\left(t\right)-H\right\}-\gamma_{P}P-\alpha_{P}P,\\ & H^{\prime }=\min\left\{\eta P,H_{c}\left(t\right)-H\right\}-\gamma_{H}\left(t\right)H-\alpha_{H}\left(t\right)H,\\ & R^{\prime }=\gamma_{P}P+\gamma_{H}\left(t\right)H, \end{array}\right. \end{array}$$

Note that people confirmed with COVID-19 may not be hospitalized due to the shortage of hospital beds, thus whether the confirmed patients could be hospitalized or not depends on the daily number of empty hospital beds. The term $\min\{\eta P,H_{c}\left(t\right)-H\}$ is used to describe the number of newly hospitalized per day to maximize hospital utilization, which is a piecewise function according to the relationship between ηP and $H_{c}\left(t\right)-H$. When $\eta P\leq H_{c}\left(t\right)-H$, namely, the hospital beds are sufficient, confirmed patients could be hospitalized with hospitalization rate η; while, when $\eta P>H_{c}\left(t\right)-H$, namely, the hospital beds are insufficient, only a part of the confirmed patients ($H_{c}\left(t\right)-H$) could be hospitalized. $H_{c}\left(t\right)$ is the number of hospital beds at time *t* in Wuhan, taking the form that is derived from the logistic model (1), as shown in Equation (3). Meanwhile, those confirmed but not hospitalized have transmissibility due to contacting others during their hospital visiting or contacting their family members during their home quarantine without absolute protective measures, θ (0≤θ≤1) is the ratio of effective contact of *P* with *S* to effective contact of *I* with *S*. See more detailed definitions and values of variables and parameters listed in Table 1.

(3) $$\begin{array}{c} H_{c}\left(t\right)=\left\{\begin{array}{cc} & H_{c_{0}},\;\;\;\;\;\;\;\;\;\;\;\;\;\;\;\;\;\;\;\;\;\;\;\;\;\;\;\;\;t\leq t_{s},\\ & \frac{H_{c_{0}}M}{H_{c_{0}}+\left(M-H_{c_{0}}\right)e^{-r(t-t0)}},\;\;\;\;t>t_{s}, \end{array}\right. \end{array}$$

where $t_{s}=13$, representing that hospital beds were offered since 23 January.

Because the medical levels, public awareness as well as the prevention and control measures were improved and strengthened gradually after 23 January, here we assumed that the contact rate c(t), the quarantine rate q(t), the diagnosis rates $\delta_{I}\left(t\right),\delta_{q}\left(t\right)$, the recovery rate $\gamma_{H}\left(t\right)$, and disease-induced death rate $\alpha_{H}\left(t\right)$ of hospitalized individuals are time-dependent functions [26]. The contact rate c(t) is assumed to be a constant before 23 January and a decreasing function with respect to time *t* after 23 January due to the lockdown strategies and raising of public awareness, which takes the following form:

(4) $$\begin{array}{c} c\left(t\right)=\left\{\begin{array}{c} c_{0},\;\;\;\;\;\;\;\;\;\;\;\;\;\;\;\;\;\;\;\;\;\;\;\;\;\;t\leq t_{1},\\ \left(c_{0}-c_{1}\right)e^{-r_{c}\left(t-t_{1}\right)}+c_{1},\;\;\;\;t>t_{1}, \end{array}\right. \end{array}$$

where $c_{0}$ is the contact rate without control measures or awareness of the disease, $c_{1}$ ($c_{1}<c_{0}$) is the minimum contact rate with the unprecedented intervention measures and self-isolation, and $r_{c}$ is the corresponding decreasing rate of the contact rate. Here, $t_{1}=13$, since the data we used for model fitting start at 10 January 2020.

The quarantined proportion q(t) is assumed to be a constant before 23 January and an increasing function with respect to time *t* due to contact tracing, which is given by

(5) $$\begin{array}{c} q\left(t\right)=\left\{\begin{array}{c} q_{0},\;\;\;\;\;\;\;\;\;\;\;\;\;\;\;\;\;\;\;\;\;\;\;\;\;\;t\leq t_{1},\\ \left(q_{0}-q_{1}\right)e^{-r_{q}\left(t-t_{1}\right)}+q_{1},\;\;\;\;t>t_{1}, \end{array}\right. \end{array}$$

where $q_{0}$ denotes the initial quarantine rate of individuals that are exposed to the virus and $q_{1}$ ($q_{1}>q_{0}$) is the maximum quarantine rate under the control measures, $r_{q}$ is the corresponding increasing rate of the quarantine rate.

The period from illness onset to be diagnosed ($\frac{1}{\delta_{I}}$) was gradually shortened due to the rising of public awareness and developing of detection technology. Thus, the diagnosis rate is a non-increasing function with respect to *t* according to the control measures that were implemented in Wuhan. Furthermore, the number of confirmed cases were increased sharply on 12 February, since the CT diagnostic method was implemented, despite the existence of the nucleic acid test method, thus we assume that the diagnosis rate reached and maintained its maximum from 12 February. Hence, the diagnosis period is described by the following piecewise function

(6) $$\begin{array}{c} \frac{1}{\delta_{I}\left(t\right)}=\left\{\begin{array}{cc} & \frac{1}{\delta_{I_{0}}},\;\;\;\;\;\;\;\;\;\;\;\;\;\;\;\;\;\;\;\;\;\;\;\;\;\;\;\;\;\;\;\;\;\;\;\;\;\;\;\;\;\;\;\;\;t\leq t_{1},\\ & \left(\frac{1}{\delta_{I_{0}}}-\frac{1}{\delta_{I_{1}}}\right)e^{-r_{\delta_{I}}\left(t-t_{1}\right)}+\frac{1}{\delta_{I_{1}}},\;\;\;\;\;\;\;\;\;\;\;\;\;\;\;\;\;t_{1}<t\leq t_{2},\\ & \frac{1}{\delta_{I_{1}}},\;\;\;\;\;\;\;\;\;\;\;\;\;\;\;\;\;\;\;\;\;\;\;\;\;\;\;\;\;\;\;\;\;\;\;\;\;\;\;\;\;\;\;\;\;t>t_{2}, \end{array}\right. \end{array}$$

where $t_{2}=33$, $\delta_{I_{0}}$ is the initial diagnosis rate of infected individuals, $\delta_{I_{1}}$ ($\delta_{I_{1}}>\delta_{I_{0}}$) is the maximum diagnosis rate of individuals, and $r_{\delta_{I}}$ is the decreasing rate of the diagnosis period from symptom onset to be diagnosed.

Similarly, the detection rate $\delta_{q}\left(t\right)$ of quarantined individuals, those that have been in contact with infected individuals and have been traced, is an increasing function from 23 January, with more detection reagents being supplied and improved detection technology. The detection rate reached a maximum on 12 February with CT diagnosis. Thus, the period from quarantine to detection is described by

(7) $$\begin{array}{c} \frac{1}{\delta_{q}\left(t\right)}=\left\{\begin{array}{c} \frac{1}{\delta_{q_{0}}},\;\;\;\;\;\;\;\;\;\;\;\;\;\;\;\;\;\;\;\;\;\;\;\;\;\;\;\;\;\;\;\;\;\;\;\;\;\;\;\;\;\;\;\;\;t\leq t_{1},\\ \left(\frac{1}{\delta_{q_{0}}}-\frac{1}{\delta_{q_{1}}}\right)e^{-r_{\delta_{q}}\left(t-t_{1}\right)}+\frac{1}{\delta_{q_{1}}},\;\;\;\;\;\;\;\;\;\;\;\;\;\;\;\;\;t_{1}<t\leq t_{2},\\ \frac{1}{\delta_{q_{1}}},\;\;\;\;\;\;\;\;\;\;\;\;\;\;\;\;\;\;\;\;\;\;\;\;\;\;\;\;\;\;\;\;\;\;\;\;\;\;\;\;\;\;\;\;\;t>t_{2}, \end{array}\right. \end{array}$$

where $\delta_{q_{0}}$ is the diagnosis rate of quarantined individuals before 23 January, $\delta_{q_{1}}$ ($\delta_{q_{1}}>\delta_{q_{0}}$) is the maximum diagnosis rate of quarantined individuals and $r_{\delta_{q}}$ is the decreasing rate of the period from quarantine to diagnosis.

For hospitalized confirmed cases, the recovery rate $\gamma_{H}\left(t\right)$ increases and the disease-induced death rate $\alpha_{H}\left(t\right)$ decreases by receiving treatment and health-care measures, with more medical supplies. Thus, $\gamma_{H}\left(t\right)$ and $\alpha_{H}\left(t\right)$ take the following form

(8) $$\begin{array}{c} \gamma_{H}\left(t\right)=\left\{\begin{array}{c} \gamma_{P},\;\;\;\;\;\;\;\;\;\;\;\;\;\;\;\;\;\;\;\;\;\;\;\;\;\;\;\;\;t\leq t_{1},\\ \left(\gamma_{P}-\gamma_{H_{1}}\right)e^{-r_{\gamma_{H}}\left(t-t_{1}\right)}+\gamma_{H_{1}},\;\;t>t_{1}, \end{array}\right. \end{array}$$

(9) $$\begin{array}{c} \alpha_{H}\left(t\right)=\left\{\begin{array}{c} \alpha_{P},\;\;\;\;\;\;\;\;\;\;\;\;\;\;\;\;\;\;\;\;\;\;\;\;\;\;\;\;\;\;\;t\leq t_{1},\\ \left(\alpha_{P}-\alpha_{H_{1}}\right)e^{-r_{\alpha_{H}}\left(t-t_{1}\right)}+\alpha_{H_{1}},\;\;\;t>t_{1}, \end{array}\right. \end{array}$$

where $\gamma_{P}$ and $\alpha_{P}$ are the recovery rate and disease-induced death rate of hospitalized individuals before 23 January, respectively, which are the same as the recovery rate and disease-induced death rate of confirmed, but not hospitalized patients, $\gamma_{H_{1}}$ ($\gamma_{H_{1}}>\gamma_{P}$) and $\alpha_{H_{1}}$ ($\alpha_{H_{1}}<\alpha_{P}$) are the maximum recovery rate and minimum disease-induced death rate of hospitalized individuals under treatment, $r_{\gamma_{H}}$ and $r_{\alpha_{H}}$ are the corresponding increasing rate of the recovery rate and the corresponding decreasing rate of the disease-induced death rate.

This model allows for us to calculate the effective reproduction number and analyze the effects of the capacity, timing and rate of supply of hospital beds on the effective reproduction number, epidemic end time, total number of confirmed cases, total number of recovered cases, and total number of deaths due to the COVID-19 outbreak in Wuhan.

### 2.3. Parameter Estimation

By using the nonlinear least-square method, we estimated the daily number of hospital beds ($H_{c}\left(t\right)$) in Wuhan after 23 January 2020 by fitting model (1) to the hospital beds data collected. When considering that the hospital beds were redundant for patients at the end of February, namely, $H_{c}\left(t\right)-H>\eta P$ always holds true from then on, the dynamics of model (2) would not be influenced by the number of hospital beds, we assumed that the number of hospital beds remained the same, as simulated in Equation (1) even though the Fangcang shelter hospitals were closed successively in March.

Furthermore, with $H_{c}\left(t\right)$ having been estimated, we fitted model (2) to the cumulative numbers of confirmed cases, recovered cases and deaths (as shown in Figure 1) to estimate unknown parameters by using the nonlinear least-square method. To obtain the confidence intervals, we assumed that the daily numbers of new confirmed, new recovered, and new deaths follow Poisson distributions with the observed data on each day being the respective means of 1000 randomly generated samples of datasets for fitting. Thus, we obtained 1000 groups of estimated parameter values, which allowed us to calculate the mean values and standard deviations of these parameters and the confidence intervals of the effective reproduction number. Note that, in our model, we fixed η=1 in agreement with the policy ‘Receive all patients’ implemented in China.

The nonlinear least-square method was carried out in MATLAB, using the command *fmincon*, which is a part of the optimization toolbox in MATLAB and finds a constrained minimum of a function of several variables. The parameter estimation by using the nonlinear least-square method in our study is to find the optimal parameter values, such that the estimated data errors with the real data are minimum. The objective function is

(10) $$\begin{array}{c} f\left(\Theta \right)=\sum_{k=1}^{k=n}{\left(\tilde{C}\left(T_{k}\right)-C\left(T_{k}\right)\right)}^{2}+\sum_{k=1}^{k=n}{\left(\tilde{R}\left(T_{k}\right)-R\left(T_{k}\right)\right)}^{2}+\sum_{k=1}^{k=n}{\left(\tilde{D}\left(T_{k}\right)-D\left(T_{k}\right)\right)}^{2} \end{array}$$

where Θ is the vector of parameters to be estimated, *n* is the length of the epidemic data period, here n=94, since we used data from 10 January to 12 April, lasting for 94 days. $T_{k}$ is the corresponding date of the k−th day since 10 January 2020. $\tilde{C}\left(T_{k}\right),\tilde{R}\left(T_{k}\right),\tilde{D}\left(T_{k}\right)$ are the actual reported cumulative numbers of confirmed cases, recovered cases and deaths on the date $T_{k}$, respectively. $C\left(T_{k}\right),R\left(T_{k}\right),D\left(T_{k}\right)$ are the estimated cumulative numbers of confirmed cases, recovered cases, and deaths on the date $T_{k}$, respectively. The dynamics of C(t),R(t),D(t) are governed by

(11) $$\begin{array}{c} \left\{\begin{array}{cc} & \frac{C(t)}{dt}=\delta_{I}\left(t\right)I+\delta_{q}\left(t\right)E_{q},\\ & \frac{R(t)}{dt}=\gamma_{P}P+\gamma_{H}\left(t\right)H,\\ & \frac{D(t)}{dt}=\alpha_{P}P+\alpha_{H}\left(t\right)H. \end{array}\right. \end{array}$$

where $I,E_{q},P,H$ are determined by model (2).

## 3. Results

### 3.1. Parameter Estimation

By using the next generation matrix method [27], the basic reproduction number could be calculated, then considering the time varying parameters, and that the ratio of the susceptible population to the total population is approximately 1, the effective reproduction number can be approximately defined [26] as

(12) $$\begin{array}{c} R_{e}\left(t\right)=\frac{(1-q(t))c(t)\beta }{\alpha_{I}+\delta_{I}\left(t\right)}\left(1+\frac{\theta \delta_{I}\left(t\right)}{\min\left\{\eta ,\frac{H_{c}\left(t\right)-H\left(t\right)}{P(t)}\right\}+\gamma_{P}+\alpha_{P}}\right)+\frac{\theta q(t)c(t)\beta }{\min\left\{\eta ,\frac{H_{c}\left(t\right)-H\left(t\right)}{P(t)}\right\}+\gamma_{P}+\alpha_{P}}. \end{array}$$

**Figure 3:**
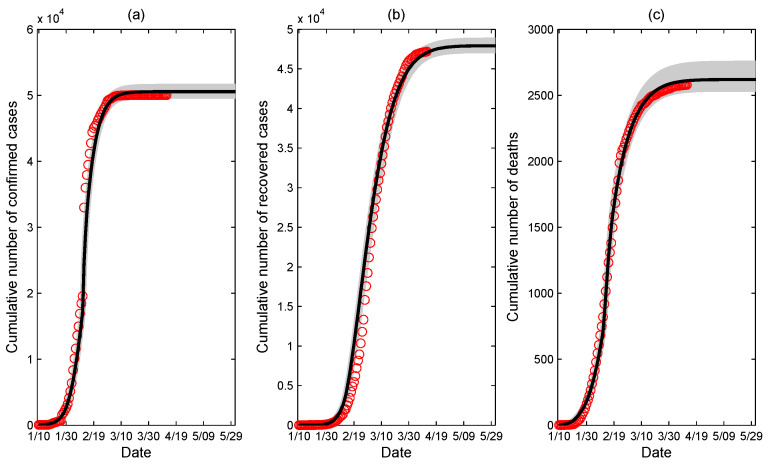
Fitting result for the data from 10 January to 12 April 2020 in Wuhan. (**a**) Fitting of cumulative number of confirmed cases; (**b**) fitting of cumulative number of recovered cases; and (**c**) fitting of cumulative number of deaths. The red circles represent the reported data, the black curves are the best fitting curves of model (2) to these data, and the gray regions are the 95% confidence intervals.

Based on the hospital bed data in Wuhan from 31 January to 25 February and model (1), which simulates the growth pattern of the hospital bed numbers with the estimated parameters *r* and *M*, we estimated the daily number of hospital beds, as shown in Figure 1d. By fitting the model (2) to the data on the cumulative numbers of confirmed cases, recovered cases, and deaths, we estimated the values of unknown parameters listed in Table 1. The fitting results are given in Figure 3, showing that the fitted model captures the data well, in which the red circles are the data from 10 January to 12 April, the black curves are the fitted curves, and the gray regions are the 95% confidence intervals.

**Figure 4:**
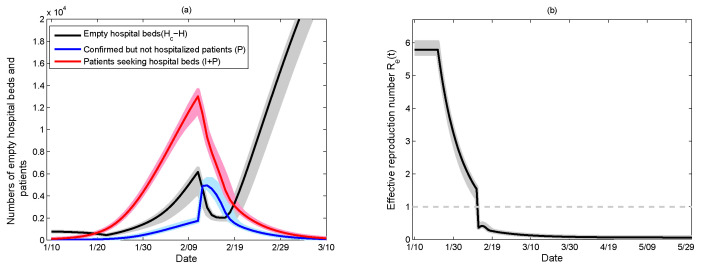
(**a**) Comparison of numbers of empty hospital beds and patients. The black, blue and red curves represent the numbers of empty hospital beds, confirmed but not hospitalized patients and patients seeking hospital beds, respectively. (**b**) Estimated effective reproduction number. The shadowed regions are the 95% confidence intervals.

The number of empty hospital beds in Wuhan ($H_{c}-H$, the black curve), the number of confirmed, but not hospitalized, patients (*P*, the blue curve) and the number of patients seeking hospital beds (I+P, the red curve) are shown in Figure 4a, from which we can see that the number of empty hospital beds is less than the confirmed but not hospitalized patients from 13 to 18 February, which is mainly because of the sudden increase of newly confirmed cases due to a change in national test standards on 12 February. Note that the number of patients seeking hospital beds is much higher than the number of empty hospital beds from 20 January to 19 February, indicating that the hospital beds were in urgent need during that period, consistent with the fact that the severe situation of a shortage of hospital beds occurred in the initial stage of the COVID-19 outbreak and the goal “hospital beds are waiting for patients” had been realized since 21 February [7].

Meanwhile, the time-dependent effective reproduction number $R_{e}\left(t\right)$ was calculated, as shown in Figure 4b, from which we can see that the effective reproduction number $R_{e}\left(t\right)$ had fallen below the threshold of 1 since 12 February. This is attributed to the increasing diagnosis rate because the clinically diagnosed cases had also been recognized as being confirmed cases since 12 February. Note that a slight rebound occurred from 13 to 18 February. This was induced by the inadequate isolation of those patients who had been confirmed, but not hospitalized, due to the shortage of hospital beds for confirmed patients.

### 3.2. Sensitivity Analysis

Sensitivity analysis of the effective reproduction number $R_{e}\left(t\right)$ calculated in (12) with respect to various parameters over time was performed by conducting Latin Hyperbolic Sampling (LHS) and evaluating the partial rank correlation coefficients (PRCCs) [31]. This allows for us to assess whether the significance of one parameter occurs over an entire time interval during the progression of the model dynamics.

**Figure 5:**
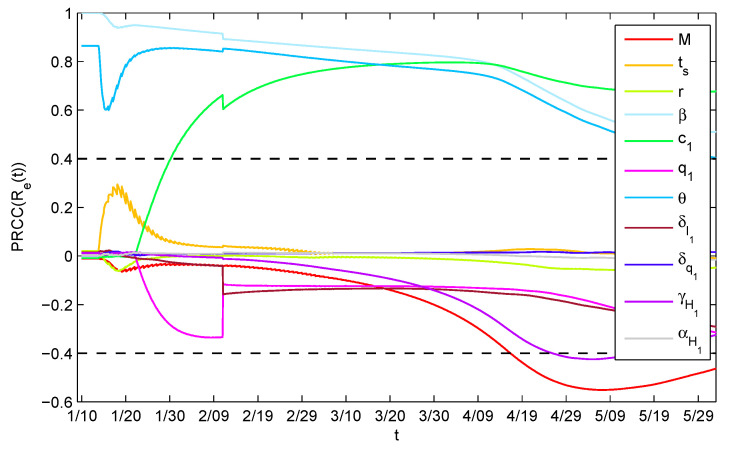
Partial rank correlation coefficients (PRCCs) of $R_{e}\left(t\right)$ for $M,t_{s},r,\beta ,c_{1},q_{1},\theta ,\delta_{I_{1}},\delta_{q_{1}},\gamma_{H_{1}},\alpha_{H_{1}}$. The Latin Hypercube Sampling was done with 10,000 bins.

Parameters are those that are related to the number of hospital beds (the hospital beds capacity *M*, the supply timing $t_{s}$, the supply rate *r*) and those related to the epidemic transmission and the control interventions (the transmission probability per contact β, the minimum contact rate $c_{1}$, the maximum quarantine rate $q_{1}$, the contact rate ratio θ of *P* to *I*, the maximum diagnosis rate of infected individuals $\delta_{I_{1}}$ and quarantined individuals $\delta_{q_{1}}$, the maximum recovery rate of hospitalized individuals with treatment $\gamma_{H_{1}}$, and the minimum disease induced-death rate of hospitalized individuals with treatment $\alpha_{H_{1}}$). The results(see Figure 5) showed that $\beta ,c_{1},\theta$ are positively correlated with $R_{e}\left(t\right)$ almost over the entire time interval. This illustrates the significant effect of mitigating disease transmission by reducing $\beta ,c_{1},\theta$, which can be realized by enhancing the intensity of wearing masks (reducing β), keeping social distancing (reducing $c_{1}$), and isolating confirmed, but not hospitalized, patients (reducing θ).

**Figure 6:**
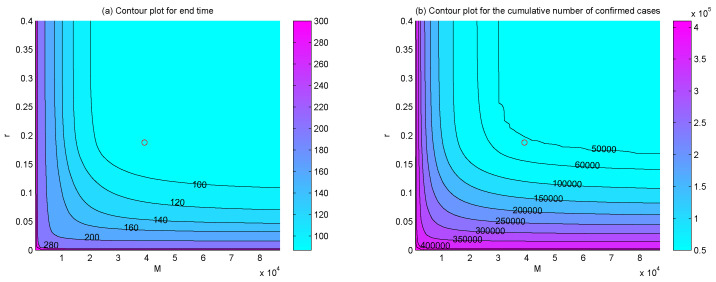

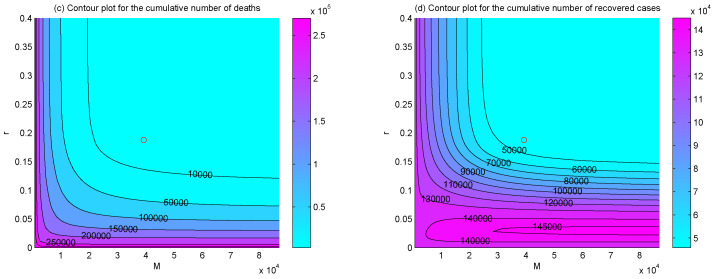
Contour plots of (**a**) the end time, (**b**) the final number of confirmed cases, (**c**) the final number of deaths, and (**d**) the final number of recovered cases with respect to *M* and *r*. The circles represent the positions of (M,r) that we have estimated.

**Figure 7:**
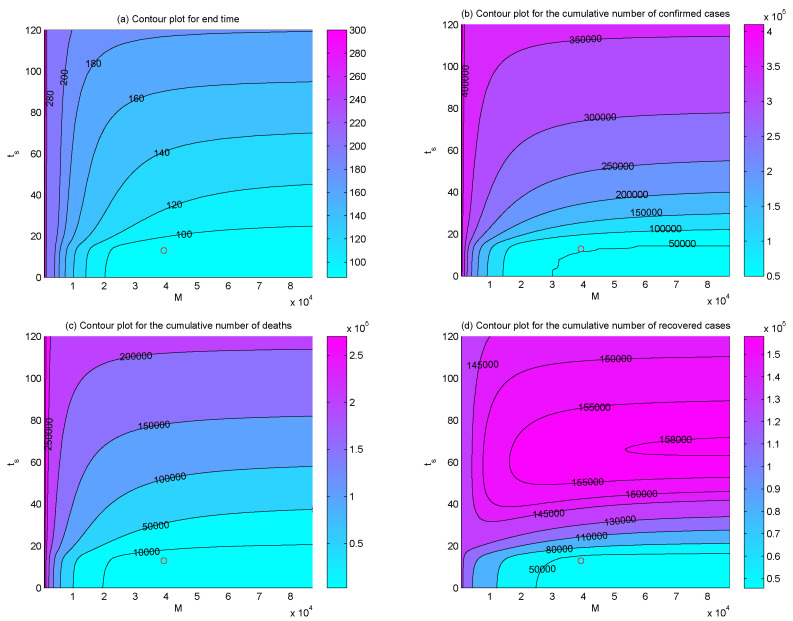
Contour plots of (**a**) the end time, (**b**) the final number of confirmed cases, (**c**) the final number of deaths, and (**d**) the final number of recovered cases with respect to *M* and $t_{s}$. The circles represent the positions of $\left(M,t_{s}\right)$ that we have estimated.

**Figure 8:**
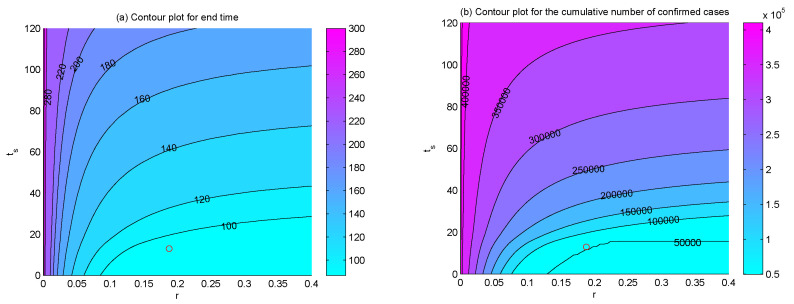

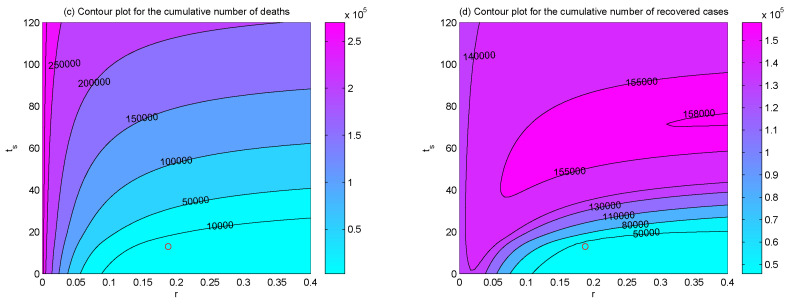
Contour plots of (**a**) the end time, (**b**) the final number of confirmed cases, (**c**) the final number of deaths, and (**d**) the final number of recovered cases with respect to *r* and $t_{s}$. The circles represent the positions of $\left(r,t_{s}\right)$ we have estimated.

To examine the dependence of the end time and the final size of the epidemic on the hospital beds capacity *M*, the supply rate *r*, and supply timing $t_{s}$, we plotted the contour plots of the end time, the total numbers of confirmed cases, recovered cases, and deaths with respect to *M* and *r* (Figure 6), *M* and $t_{s}$ (Figure 7), *r* and $t_{s}$ (Figure 8), respectively. The results indicated that, if the hospital beds supply time $t_{s}$ was fixed, namely, the supply of hospital beds dedicated to COVID-19 patients was started on 23 January, increasing hospital beds capacity *M* and supply rate *r* would bring forward the end time of the epidemic (Figure 6a), the final size of confirmed cases and final number of deaths would be reduced (Figure 6b,c). Similarly, if the supply rate *r* or the maximum number of hospital beds *M* was fixed as estimated, the end time of the epidemic would be brought forward (Figure 7a and Figure 8a), the final cumulative number of confirmed cases (Figure 7b and Figure 8b), and the final number of deaths (Figure 7c and Figure 8c) would be reduced greatly with more hospital beds (increasing *M*) or a faster supply rate (increasing *r*), and earlier interventions of supplementing hospital beds (decreasing $t_{s}$).

It is worth mentioning that the effectiveness of supplying hospital beds on shortening the outbreak duration, reducing the final size of confirmed and deaths are only significant when parameters $M,r,t_{s}$ vary in proper ranges. Out of the ranges, increasing M,r or reducing $t_{s}$ would have no impact. What is more, the final size of the recovered population may increase at first and then decrease with the increasing of *r*, *M* and $t_{s}$. For example, we can see that when *M* = 40,000 (Figure 6d and Figure 7d) or $t_{s}$ = 40 (Figure 7d and Figure 8d), the final numbers of recovered cases increase and then decrease with the increasing of $r,t_{s}$ or $M,t_{s}$, which means that there may exist an appropriate combination of $M,r,t_{s}$ in order to ensure fewer confirmed cases and deaths and more recovered cases. Thus, it would be interesting to consider if there is an optimal strategy of supplying hospital beds to maximize the final size of the recovered population and minimize the final number of confirmed cases and deaths with minimum expenditure, which will fall within the scope of our future work.

**Figure 9:**
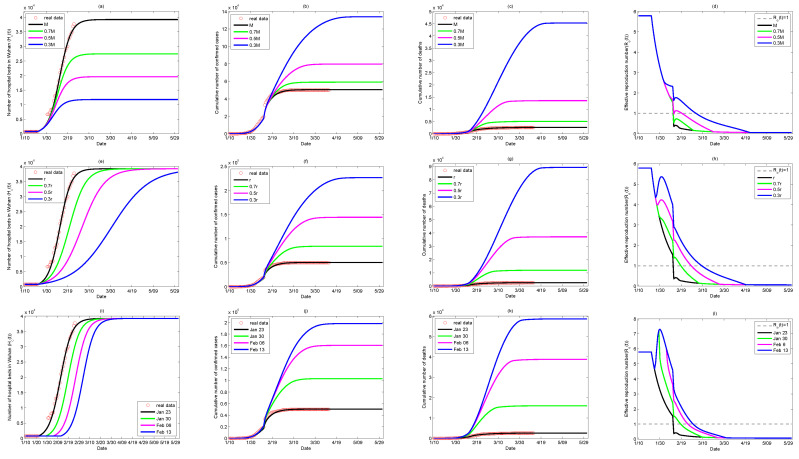
Impact of hospital beds. Impact of the maximum capacity of hospital beds *M* on: (**a**) the number of hospital beds, (**b**) the cumulative number of confirmed cases, (**c**) the cumulative number of deaths, and (**d**) the effective reproduction number; impact of the increasing rate of hospital beds *r* on: (**e**) the number of hospital beds, (**f**) the cumulative number of confirmed cases, (**g**) the cumulative number of deaths, and (**h**) the effective reproduction number; impact of the intervention time $t_{s}$ of supplying hospital beds on: (**i**) the number of hospital beds, (**j**) the cumulative number of confirmed cases, (**k**) the cumulative number of deaths, and (**l**) the effective reproduction number.

We investigated how the cumulative number of confirmed cases and the cumulative number of deaths vary with different values of the hospital beds capacity *M*, the hospital beds supply rate *r*, and the intervention time of expropriated hospital beds $t_{s}$ in order to further explore the exact impact of medical resources on the COVID-19 outbreak in Wuhan. We conducted a sensitivity analysis to show what would happen if the total number of hospital beds *M* provided was reduced by 30%,50%,70%, or the hospital beds supply rate *r* was reduced to 0.7r,0.5r,0.3r, or the supply of hospital beds dedicated to COVID-19 patients was implemented one week later (from 30 January), two weeks later (from 6 February), and three weeks later (from 13 February), respectively, as shown in Figure 9. The results illustrated that reducing the total number of hospital beds (decreasing *M*), slowing down the establishment or designation rate of new hospitals (decreasing *r*), and delaying the intervention time (increasing $t_{s}$) all lead to severer outbreaks with significant increases of the cumulative number of confirmed cases and the cumulative number of deaths, and longer durations of COVID-19 in Wuhan.

Meanwhile, it follows from Figure 9d,h,l that the effective reproduction number is obviously affected by the total number of hospital beds, the increasing rate of the availability of hospital beds, and the time when the hospital beds are supplied. We can observe that reducing the supplementation rate of hospital beds, or reducing the maximum number of hospital beds, may not affect the general trend (i.e., decreasing) of the effective reproduction number. However, all of them, more or less, could magnify the value of the effective reproduction number in some period and postpone the time when the threshold value is reduced to 1. For example, a significant increase of $R_{e}\left(t\right)$ occurred when the supplying rate of hospital beds was reduced to 0.3r or the supplying hospital beds was implemented three weeks later (on 13 February ), which would result in larger outbreaks.

In order to investigate the important role of Huoshenshan, Leishenshan, and Fangcang shelter hospitals, we only estimated the increasing rate and capacity of hospital beds in designated hospitals. Fitting model (1) by using the number of beds in designated hospitals shown in Figure 1d (the orange bars), we obtained that r=0.1814,M=22,010. The supply rate and capacity of hospital beds are both reduced without Huoshenshan, Leishenshan, and Fangcang shelter hospitals. In Table 2, we summarized how accurately the length of the epidemic, the length of controllable time ($R_{e}\left(t\right)<1$ for the first time), the final confirmed cases, and the final dead cases vary with respect to different hospital beds capacity, supply rate, and supply timing. We have to emphasize that the results demonstrated that, if Huoshenshan, Leishenshan, and Fangcang shelter hospitals were not established, 73,126 confirmed cases (instead of 50,340 cases in reality) and 10,393 deaths (instead of 3869 deaths in reality) would be reported in Wuhan. Thus, the establishment of Huoshenshan, Leishenshan, and Fangcang shelter hospitals avoided 22,786 persons from being infected and saved 6524 lives. If the government had implemented the intensive interventions without providing any additional hospital beds, the final number of confirmed cases would have been 412,700, and the final number of deaths would have been 278,460, illustrating that the designation and establishment of hospital avoided 362,360 persons from infection and saved the lives of 274,591 people.

## 4. Discussion

The global outbreak of COVID-19 caused by a new strain of coronaviruse has had profound impacts on almost all of the world and it has become the most serious respiratory virus since the 1918 H1N1 influenza pandemic. Such a pandemic has put a very considerable strain on public health organizations and led to significant shortages in health-care resources. Therefore, for preserving public health, it is of major importance to quantify how medical resource availability affects the COVID-19 outbreak and so the experience gained in fighting against COVID-19 in Wuhan, China, could provide a model for other countries and other infectious diseases in the future.

We proposed a piecewise smooth model (2) that was based on the transmission progression of COVID-19 and the control policy implemented in Wuhan, China, in order to describe the limitation of hospital beds and investigated how the capacity, the supply rate and the supply timing affected the COVID-19 outbreak in Wuhan. With the strengthening of control measures, we modeled the epidemic system and its parameters by assuming that they are related by a piecewise function with respect to time and that the number of hospital beds follows a logistic growth curve. By using the data on hospital beds provided for COVID-19 patients in Wuhan from 31 January to 25 February, the daily number of hospital beds was estimated by fitting the data to the logistic model (1) with estimated values of *r* and *M*. Subsequently, by employing the nonlinear least-square method to fit model (2) with 1000 samples of the data, including the cumulative numbers of confirmed cases, recovered cases and deaths generated from Poisson process, the mean values, and standard deviations were estimated. The simulation results showed that the number of empty hospital beds were not enough for patients seeking hospital beds from 20 January to 19 February (Figure 4), which is consistent with the fact that a shortage of hospital beds was a severe problem of Wuhan in the early phase of the outbreak and the severe situation was relieved by 21 February.

The PRCCs of the effective reproduction number $R_{e}\left(t\right)$ with respect to various parameters over time (Figure 5) showed that, reducing θ, which can be realized by isolating confirmed but not hospitalized patients completely by supplying more hospital beds, could significantly reduce the effective reproduction number $R_{e}\left(t\right)$ and, thus, lower the transmission risk of COVID-19 in Wuhan. The sensitivity analysis (Figure 6, Figure 7, Figure 8 and Figure 9) also identified the vital role of the supply capacity, supply rate and supply timing of hospital beds. The findings demonstrated that reducing the total number of hospital beds, slowing down their supply rate and delaying the timing of supplementing hospital beds, all aggravate the outbreak severity, including magnifying the cumulative numbers of confirmed cases and deaths and prolonging the period of the outbreak in Wuhan. Meanwhile, lack of urgency in supplying hospital beds also enlarges the value of the effective reproduction number during the outbreak and postpones the time when the threshold value is reduced to 1. These findings suggest that a shortage of hospital beds is an important issue that must not be neglected. Even with the prompt and unprecedented strong control measures, such as lockdown of cities, travel restriction, contact tracing, etc., insufficient hospital beds would impede the mitigation of the outbreak and cause a severer situation.

The results that are presented in Table 2 revealed that the quick establishment of the Huoshenshan and Leishenshan Hospitals in a short time and the deploying of Fangcang shelter hospitals contributed to the rapidly increased numbers of hospital beds in Wuhan, which relieved the medical pressure and helped curbing the COVID-19 outbreak in Wuhan. They avoided 22,789 infections and saved 6524 lives in Wuhan. Furthermore, the timely intervention of designating hospitals and establishing Huoshenshan, Leishenshan, and Fangcang shelter hospitals avoided 362,360 infections and saved the lives of 274,591 people in Wuhan.

We have to point out that the results of contour plots revealed an interesting problem, i.e., there may exist an optimal strategy of supplementing hospital beds, with an optimal chosen capacity, supply rate, and supply timing, in order to ensure that the final size of the infected population is low and that the expenditure is economical, a topic for our future work. Note that in this paper, the asymptomatic population was not considered, and also, it would be more reasonable to distinguish patients with mild symptoms from patients with severe symptoms, which is also a subject for our further work. Despite this, a shortage of medical resources may increase the mortality rate and reduce the recovery rate, which would be considered in our future work as well. Furthermore, the outbreak of COVID-19 must have diverted the attention of medical resources from other diseases (for example, flu, common pneumonia, etc.), so it would also be interesting to consider the competition for medical resources between COVID-19 and other diseases.

We focus on the situation in Wuhan, China, but the model can be extended in order to describe the shortage of medical resources in other countries by considering the control policy they implemented. For example, when studying the case in India, it would be reasonable to consider the detection rate as a piecewise function, since the capacity of detection per day there is limited.

## 5. Conclusions

This paper presents the first use of a logistic model fitted to the public published data of the designated hospital beds, Huoshenshan, Leishenshan and Fangcang shelter hospital beds to describe the supply of hospital beds in Wuhan by using only three parameters (the hospital beds capacity *M*, the supply rate *r*, and the supply timing $t_{s}$). It is also the first use of a piecewise smooth system to describe the situation whereby confirmed patients had no access to hospitals due to a lack of hospital beds in Wuhan. Multi-source data, including the hospital beds data and the epidemic data, were used to conduct retrospective studies in order to analyze the impact of hospital beds. Our results strongly support the importance of timely establishment of the Huoshenshan and Leishenshan hospitals and Fangcang shelter hospitals that saved thousands of lives. These results could provide suggestions for policy-makers in other countries.

## Figures and Tables

**Table 1:** Definition and values of variables and parameters for system (2).

Variables		Description	Initial Value	Resource
*S*		Susceptible population	11,081,000	[23]
*E*		Exposed population	73.9089 (0.7779)	LS
*I*		Infected population	102.9589 (0.9503)	LS
$S_{q}$		Quarantined susceptible population	739	[24]
$E_{q}$		Quarantined exposed population	0.9964 (0.1085)	LS
*P*		Confirmed but not hospitalized population	0	data
*H*		Confirmed and hospitalized population	38	data
*R*		Recovered population	2	data
Parameters		Description	Mean(std)	Resource
c(t)	$c_{0}$	Contact rate before 23 Jan	12.3548 (0.0291)	LS
	$c_{1}$	Minimum contact rate with control strategies	0.7703 (0.0184)	LS
	$r_{c}$	Exponential decreasing rate of the contact rate	0.0553 (0.0009)	LS
β		Transmission probability from *I* to *S* per contact	0.0521 (0.0002)	LS
θ		Effective contact ratio of *P* with *S* to *I* with *S*	0.4842 (0.0051)	LS
q(t)	$q_{0}$	Quarantine rate before 23 Jan	$4.5\times 10^{-5}$ ($4.3\times 10^{-7}$)	LS
	$q_{1}$	Maximum quarantine rate with control measures	0.1046 (0.0024)	LS
	$r_{q}$	Exponential increasing rate of quarantine rate	0.1372 (0.0062)	LS
λ		Releasing rate of quarantined susceptibles	1/14	[26]
σ		Progression rate of exposed individuals to infectives	1/5.2	[28,29,30]
$\delta_{I}\left(t\right)$	$\delta_{I_{0}}$	Diagnosis rate of infected individuals before 23 Jan	0.1154 (0.0011)	LS
	$\delta_{I_{1}}$	Maximum diagnosis rate of infected individuals	0.8382 (0.0033)	LS
	$r_{\delta_{I}}$	Exponential decreasing rate of diagnosis period from symptom onset to detection	0.0154 (0.0024)	LS
$\alpha_{I}$		Disease-induced death rate of *I*	0.0017 ($6.1\times 10^{-5}$)	LS
$\delta_{q}\left(t\right)$	$\delta_{q_{0}}$	Diagnosis rate of quarantined individuals before 23 Jan	0.0906 (0.0044)	LS
	$\delta_{q_{1}}$	Maximum diagnosis rate of quarantined individuals	0.8093 (0.0068)	LS
	$r_{\delta_{q}}$	Exponential decreasing rate of diagnosis period from quarantine to detection	0.2213 (0.0169)	LS
η		Hospitalization rate	1	Policy
$\gamma_{H}$	$\gamma_{P}$	Recovery rate of confirmed individuals without hospitalization	0.0098 (0.0012)	LS
	$\gamma_{H_{1}}$	Maximum recovery rate of hospitalized individuals with treatment	0.2394 (0.003)	LS
	$r_{\gamma_{H}}$	Exponential increasing rate of recovery rate	0.006 ($1.6531\times 10^{-4}$)	LS
$\alpha_{H}$	$\alpha_{P}$	Disease-induced death rate of confirmed individuals without hospitalization	0.025 ($5.6958\times 10^{-4}$)	LS
	$\alpha_{H_{1}}$	Minimum disease-induced death rate of hospitalized individuals with treatment	0.0011 ($2.8338\times 10^{-5}$)	LS
	$r_{\alpha_{H}}$	Exponential decreasing rate of disease-induced death rate	0.1801 (0.0015)	LS
$H_{c}\left(t\right)$	$H_{c_{0}}$	Initial number of hospital beds for COVID-19 in Wuhan	800	data
	*r*	Net increasing rate of hospital beds	0.1877	LS
	*M*	Maximum capacity of hospital beds	39274	LS

**Table 2:** The impact of hospital beds on the length of the epidemic, the length it takes from 10 January to the time when $R_{e}\left(t\right)=1$, the final number of confirmed cases, and final number of deaths from COVID-19 in Wuhan.

Parameters	Final Confirmed Cases	Final Deaths	Length of the Epidemic	Length of $R_{e}\left(t\right)<1$
Baseline values	50,537	2615	105	33
0.7 M	59,144	5100	110	34
0.5 M	79,844	13,565	124	42
0.3 M	134,070	45,333	154	53
0.7r	84,148	11,947	117	41
0.5r	144,390	37,089	133	50
0.3r	226,890	89,358	156	60
$t_{s}$+7	103,230	16,039	119	40
$t_{s}$+14	160,570	38,814	132	47
$t_{s}$+21	198,230	58,667	139	52
No Huoshenshan, Leishenshan, Fangcang hospitals	73,126	10,393	119	38
No hospital supplementation	412,700	278,460	326	69

## Abbreviations

The following abbreviations are used in this manuscript:

WHO	World Health Organization
LHS	Latin Hyperbolic Sampling
PRCCs	Partial rank correlation coefficients

## References

[1] World Health Organization https://www.who.int/health-topics/coronavirus.

[2] Zhou P., Yang X., Wang X., Hu B., Zhang L., Zhang W., Si H., Zhu Y., Li B., Huang C. (2020). A pneumonia outbreak associated with a new coronavirus of probable bat origin. Nature.

[3] Lu R., Zhao X., Li J., Niu P., Yang B., Wu H., Wang W., Song H., Huang B., Zhu N. (2020). Genomic characterisation and epidemiology of 2019 novel coronavirus: Implications for virus origins and receptor binding. Lancet.

[4] Epidemiology Working Group for NCIP Epidemic Response (2020). The epidemiological characteristics of an outbreak of 2019 novel coronavirus diseases (COVID-19) in China. Chin. J. Epidemiol..

[5] ChinaDaily https://www.chinadaily.com.cn/a/202002/09/WS5e3fba1ca3101282172760aa.html.

[6] Chen S., Zhang Z., Yang J., Wang J., Zhai X., Barnighausen T., Wang C. (2020). Fangcang shelter hospitals: A novel concept for responding to public health emergencies. Lancet.

[7] Wuhan Government http://www.wh.gov.cn/sy/whyw/202003/t20200316_961242.shtml.

[8] Wu J.T., Leung K., Leung G.M. (2020). Nowcasting and forecasting the potential domestic and international spread of the 2019-nCoV outbreak originating in Wuhan, China: A modelling study. Lancet.

[9] Tang B., Wang X., Li Q., Bragazzi N.L., Tang S., Xiao Y., Wu J. (2020). Estimation of the Transmission Risk of the 2019-nCoV and Its Implication for Public Health Interventions. J. Clin. Med..

[10] Hao X., Cheng S., Wu D., Wu T., Lin X., Wang C. (2020). Reconstruction of the full transmission dynamics of COVID-19 in Wuhan. Nature.

[11] Maier B., Brockmann D. (2020). Effective containment explains subexponential growth in recent confirmed COVID-19 cases in China. Science.

[12] Hsiang S., Allen D., Annan-Phan S., Bell K., Bolliger I., Chong T., Druckenmiller H., Huang L.Y., Hultgren A., Krasovich E. (2020). The effect of large-scale anti-contagion policies on the COVID-19 pandemic. Nature.

[13] Worby C., Chang H. (2020). Face mask use in the general population and optimal resource allocation during the COVID-19 pandemic. Nat. Commun..

[14] Tian H., Liu Y., Li Y., Wu C., Chen B., Kraemer M., Li B., Cai J., Xu B., Yang Q. (2020). An investigation of transmission control measures during the first 50 days of the COVID-19 epidemic in China. Science.

[15] Rong X., Liu Y., Chu H., Zhou L., Chen M., Fan M., Zhu H. (2020). Effect of Healthcare Staff on the Precention and Control of COVID-19. ACTA Math. Appl. Sin..

[16] Sun G., Wang S., Li M., Li L., Zhang J., Zhang W., Jin Z., Feng G. (2020). Transmission dynamics of COVID-19 in Wuhan, China: Effects of lockdown and medical resources. Nonlinear Dynam..

[17] Li J., Yuan P., Heffernan J., Zheng T., Ogden N., Sander B., Li J., Li Q., Belair J., Kong J.D. (2020). Fangcang shelter hospitals during the COVID-19 epidemic, Wuhan, China. B. World Health Organ..

[18] Wang W., Ruan S. (2004). Bifurcation in an epidemic model with constant removal rate of the infectives. J. Math. Anal. Appl..

[19] Wang W. (2006). Backward bifurcation of an epidemic model with treatment. Math. Biosci..

[20] Wang A., Xiao Y., Zhu H. (2018). Dynamics of a Filippov epidemic model with limited hospital beds. Math. Biosci. Eng..

[21] Wang A., Xiao Y., Smith R. (2019). Multiple equilibria in a non-smooth epidemic model with medical-resource constraints. Bull. Math. Biol..

[22] National Health Commission of the People’s Republic of China http://en.nhc.gov.cn/index.html.

[23] Health Commission of Hubei Province http://wjw.hubei.gov.cn/.

[24] Wuhan Municipal Health Commission http://wjw.wuhan.gov.cn/.

[25] The Use of Designated Hospital Beds in Wuhan. http://wjw.wuhan.gov.cn/ztzl_28/fk/tzgg/202004/t20200430_1197315.shtml.

[26] Tang B., Xia F., Tang S., Bragazzi N.L., Li Q., Sun X., Liang J., Xiao Y., Wu J. (2020). The effectiveness of quarantine and isolation determine the trend of the COVID-19 epidemics in the final phase of the current outbreak in China. Int. J. Infect. Dis..

[27] Driessche P., Watmough J. (2002). Reproduction numbers and sub-threshold endemic equilibria for compartmental models of disease transmission. Math. Biosci..

[28] Chinese Center for Disease Control and Prevention http://2019ncov.chinacdc.cn/2019-nCoV/.

[29] Backer J.A., Klinkenberg D., Wallinga J. (2020). Incubation period of 2019 novel coronavirus (2019-nCoV) infections among travellers from Wuhan, China, 20–28 January 2020. Eurosurveillance.

[30] Li Q., Guan X., Wu P. (2020). Early transmission dynamics in Wuhan, China, of novel coronavirus—infected pneumonia. New Engl. J. Med..

[31] Marino S., Hogue I.B., Ray C.J., Kirschner D.E. (2008). A methodology for performing global uncertainty and sensitivity analysis in systems biology. J. Theor. Biol..

